# Predictors of Low Birth Weight at Lumbini Provincial Hospital, Nepal: A Hospital-Based Unmatched Case Control Study

**DOI:** 10.1155/2020/8459694

**Published:** 2020-03-26

**Authors:** Saneep Shrestha, Sandeep Shrestha, Upasana Shakya Shrestha, Kamala Gyawali

**Affiliations:** ^1^Department of Community Medicine, Universal College of Medical Sciences, Ranigaun Bhairawaha, Nepal; ^2^Department of Pediatrics, Universal College of Medical Sciences, Ranigaun Bhairawaha, Nepal; ^3^Adolescent Empowerment Specialist, CARE Nepal, Laxminagar, Butwal, Nepal; ^4^Nursing Coordinator, Lumbini Provincial Hospital, Hospital Road, Butwal, Nepal

## Abstract

**Background:**

Low birth weight (LBW) is defined as the birth weight of live born infants below 2500 g, regardless of gestational age. It is a public health problem caused by factors that are potentially modifiable. The purpose of this study was to determine the socioeconomic, obstetric, and maternal factors associated with LBW in Lumbini Provincial Hospital, Nepal.

**Methods:**

The study was conducted using case control study design with 1 : 2 case control ratio. A total of 105 cases and 210 controls were taken in this study. Data were entered on Epi data software version 3.1 and exported to Statistical Package for Social Science (SPSS) software version 25 for analysis. Characteristics of the sample were described using mean and standard deviation. Bivariate analysis was done to assess the association between dependent and independent variables. The ultimate measure of association was odds ratio. Variables found to be associated with bivariate analysis were entered into a multivariable logistic regression model to identify predictors of LBW.

**Results:**

The mean age of the participants was 25.98 years with ±4.40 standard deviation. Mothers with literate educational background (AOR 0.32, 95% CI 0.13–0.81), housewife (AOR 2.63, 95% CI 1.11–6.20), vaginal mode of delivery (AOR 0.45, 95% CI 0.25–0.82), gestational age <37 weeks (AOR 2.51, 95% CI 1.15–5.48), history of LBW (AOR 5.12, 95% CI 1.93–13.60), and maternal weight <50 kilograms (AOR 2.23, 95% CI 1.23–4.02) were significantly associated with LBW.

**Conclusion:**

Educational and occupational status, mode of delivery, gestational age, maternal weight, and history of LBW were found to be independent predictors of LBW. There is need of developing coordination with education sector for increasing educational status of mothers and adolescent girls. Social determinants of health need to be considered while developing interventional programs. Similarly, interventional programs need to be developed considering identified predictors of low birth weight.

## 1. Background

The World Health Organization (WHO) defines low birth weight (LBW) as the birth weight of live born infants below 2500 g, regardless of gestational age [1]. Global prevalence of low birth weight is estimated to be 14.6% with prevalence varying across regions from 7.2% in developed regions to 17.3% in Asia and within region from 5.6% in central Asia to 27.2% in Southern Asia [2]. Information regarding birth weight is of great importance as it is an indirect indicator of maternal nutrition and predictive indicator of potential neonatal death and malnutrition if the child survives [3]. LBW babies have higher probability of dying within the first month of life or associated with adverse health outcomes like stunted growth [4], delayed motor and social development or learning disabilities [5], lower IQ [6], and high mortality [7]. Also LBW shares higher proportion of global neonatal mortality which is estimated to be around 60–80% [8].

Risk factors for low birth weight are mainly associated with socioeconomic and maternal factors. Socioeconomic factors associated with low birth weight include place of residence, occupation, educational status, and wealth index [9]. Similarly, maternal factors associated with low birth weight include preterm delivery, history of low birth weight, maternal age, height, Hb level, iron supplementation, and frequency of Ante Natal Care (ANC) visit [9–12].

Previous studies conducted in Nepal have shown that the prevalence of low birth weight ranges from 9.4% to 21.6% [13–15]. Furthermore, the UNICEF and WHO estimate prevalence of low birth weight in Nepal to be 21.8% which is third highest in the world [2]. Despite significant efforts made from the Nepal health sector, prevalence of LBW remains to be high and there has been no change in prevalence of LBW between 2011 and 2016 as per the Nepal Demographic and Health Survey report. Also study conducted on LBW using case control design is limited in country settings of Nepal. Therefore, this study was mainly conducted to identify the socioeconomic, obstetric, and maternal factors associated with low birth weight. This study also aims to provide valuable information for other researchers, health service providers, and policy makers in developing interventional programs for achieving global nutrition third target of 30% reduction of low birth weight by 2025 [16].

## 2. Materials and Methods

### 2.1. Study Design and Setting

Institutional-based case control study was conducted at Lumbini Provincial Hospital, located at Butwal, Lumbini zone, Nepal, from June to July 2019. The hospital was established in 1967 and named as Lumbini zonal hospital and later upgraded to Lumbini Provincial Hospital in 2019. Currently, the catchment population of the hospital is four million and serves to thirteen districts of the western part of Nepal.

### 2.2. Study Population

Postpartum mothers who recently delivered live newborn babies were taken as study population. A case was defined as a mother who delivered a baby weighing less than 2500 g in Lumbini Provincial Hospital between 1^st^ June and 31^st^ July 2019. Similarly, control was defined as a mother who delivered a baby weighing at least 2500 g and not exceeding 4000 g in the same health facility and time frame. Newborn baby fulfilling the definition of case and control and mothers giving informed consent was included in the study. Similarly, in this study, stillbirths and newborn baby with congenital abnormalities was set as exclusion criteria.

### 2.3. Sample Size and Sampling Techniques

The sample size was calculated using Open Epi Version 3.01 statistical software for unmatched case control study. Basic parameters assumed for calculating sample size was 95% confidence level, power of study 80%, ratio of control to case 2 : 1, minimum detectable odds ratio of two, and proportion of control exposed 35% [17]. The calculated sample size was 100 cases and 200 controls. Additional 5% sample was taken for adjustment of nonresponse and missing data giving final sample size of 105 cases and 210 controls. Age and sex matching was done during selection of controls. Two controls were selected on the same day when a case was found, and controls were selected randomly for more than two eligible controls.

### 2.4. Data Collection Tool and Measurement

Data were collected using a structured questionnaire which was prepared after reviewing similar studies conducted in Nepal and other similar country settings. The questionnaire consisted of sociodemographic, socioeconomic, and obstetric information. Obstetric information was collected by reviewing the obstetric records of the participants. One-day training was provided to two hospital staff having bachelor's degree in nursing educational background for data collection procedure. All the filled questionnaires were reviewed and checked for errors by the principal investigator on regular basis.

### 2.5. Data Processing and Analysis

Data were entered on Epi data software version 3.1 and exported to Statistical Package for Social Science (SPSS) software version 25 for analysis. Characteristics of the sample were described using mean and standard deviation. Bivariate analysis was carried out to assess the association between dependent and independent variables. The ultimate effect measure (measure of association) was odds ratio, and 95% confidence intervals were used to determine statistical significance. Variables found to be associated with bivariate analysis were entered into the multivariate logistic regression model to identify predictors of LBW.

### 2.6. Ethical Consideration

Ethical approval was obtained from the Universal College of Medical Science and Teaching Hospital Institutional Review Committee (UCMS/IRC/067/19). Permission was also obtained from the Medical Superintendent and Obstetric Department of Lumbini Provincial Hospital to access the participants and their records. The study was explained to participants, and written informed consent with sign or thumb print was obtained for data collection.

## 3. Results

### 3.1. Sociodemographic Characteristics of Respondents

Table 1 shows the sociodemographic characteristics of mothers who delivered at Lumbini Provincial Hospital between 1^st^ June and 31^st^ July 2019. A total of 315 mothers (105 cases and 210 controls) participated in the study. The mean age of mothers was found to be 25.98 years with ±4.40 standard deviation. Among study participants, 227 (72.1%) mothers were of the age group of 20–30 years, 169 (53.7%) were of upper caste, 281 (89.2%) were literate, 188 (59.7%) were living in nuclear family, and 250 (79.4%) were housewives.

### 3.2. Maternal and Obstetric Characteristics of Respondents

Table 2 shows the maternal and obstetric characteristics of mothers. Mode of child delivery was vaginal (198 (62.9%). Parity of mothers was almost equal with 161 (51.1%) primiparity.

Proportion of mothers with a history of abortion and low birth weight was 28 (8.9%) and 24 (7.6%), respectively. Preterm delivery was prevalent in 48 (15.2%) mothers. Proportion of mothers with ANC visit of 4 or more was 251 (79.7%). Mean weight and hemoglobin level of mothers were found to be 54.53 kg and 10.20 g/dl with a standard deviation of ±6.69 and ± 1.19, respectively. Proportion of mothers with weight more than or equal to 50 kg was 240 (76.2%) and hemoglobin level more than 11 g/dl was 72 (22.9%). Majority of women were not having adequate amount of rest and sleep during day and night time. Proportion of women with rest time of more than 2 hours in day time and ≥8 hours in night time was 39 (12.4%) and 62 (19.7%), respectively.

### 3.3. Sociodemographic Predictors of LBW

Table 3 shows sociodemographic predictors of low birth weight. Results from bivariate analysis revealed mother's age, ethnicity, educational status, occupation, and wealth index as significant variables to be associated with low birth weight which was further entered into the multivariate regression analysis model for confounding adjustment. Results from the multivariate regression analysis model identified educational status and occupation of mothers as sociodemographic predictors of low birth weight. Literate mothers were found to have protective effect for LBW (AOR 0.32, 0.13–0.81). Similarly, the odds of having LBW infants were high among mothers whose occupation was labor or wage worker (AOR 5.21, CI 1.13–23.88) and were unemployed (AOR 2.63, CI 1.11–6.20).

### 3.4. Maternal and Obstetric Predictors of LBW

Table 4 shows maternal and obstetric determinants of LBW. Factors associated with LBW, resulting from bivariate analysis, were entered into the multivariate regression analysis model which identified mode of delivery, gestational age, history of LBW, and maternal weight as maternal and obstetric predictors of LBW. Odds of LBW increased significantly with decrease in gestational age (AOR 2.51, CI 1.15–5.48) and maternal weight (AOR 2.23, CI 1.23–4.02). Similarly, odds of having LBW with cesarean delivery were twice (AOR 2.19, CI 1.21–3.97) and high among mothers with a previous history of LBW (AOR 5.12, CI 1.93–13.60).

## 4. Discussion

This study aimed at determining the sociodemographic, maternal, and obstetric predictors of low birth weight. Literate mothers were 68% less likely to have low weight babies compared to their illiterate counterpart. This finding is consistent with other studies where literate mothers were found to have a protective effect against LBW [18–21]. Mothers involved in labor work were five times and mothers who were unemployed or housewives were two times more likely to deliver LBW baby than the employed counterpart. This finding aligns with the study conducted in Lithuania, Vientiane, and Nepal [22–24] which revealed that mother's employment status is significantly associated with LBW. The role of education and employment on infant birth weight might be due to various interactions among social determinants of health. Mothers with low educational attainment are prone to low health seeking behavior and more likely to be unemployed which further can lead to deprivation of nutritious food, good housing condition, and wealth which are found to be independent risk factors of LBW [19, 25–27].

The odds of delivering LBW baby among mothers with a previous history of LBW were five times higher than that of mothers with no previous history of LBW. This finding is similar to the study conducted in Ethiopia [10]. Similarly, odds of delivering LBW baby were twice among mothers with preterm delivery compared to mothers who delivered at term pregnancy. This finding parallels with the previous randomized control trial study conducted in Kenya [28], cohort study conducted in Ethiopia [29], and case control study conducted in Ghana [30], Ethiopia [11, 31], and Nepal [32]. In this study, significant association was observed between mode of delivery and LBW with odds of LBW among cesarean delivery being twice compared to vaginal delivery. The study findings align with the previous studies conducted in Kenya [28], Spain [33], Brazil [34], and Iran [35]. The observed association should be understood cautiously as maternal conditions of having twins, with a gestational age of less than 37 weeks, are more prone to cesarean delivery and are found to be contributing factors for LBW [10, 34]. However, no significant association was observed between mode of delivery and LBW in a study conducted in Nepal [13]. In this study, low maternal weight was found to be an independent risk factor for LBW which is supported by findings of previous study conducted in India [36, 37] and Ethiopia [38] using case control and cross-sectional study design, respectively.

Regular ANC during pregnancy is beneficial for both the pregnant mother and developing baby as obstetric complications can be identified during ANC and managed timely [18]. Studies conducted in Malawi and Uganda [39], Ethiopia [29], and Bangladesh [40] have identified an inadequate number of ANC visits to be associated with higher odds of LBW. However, no significant association was observed between the number of ANC visits and LBW in our study.

Despite extensive endeavors made in the study, it could not avert three basic limitations. First, the study findings cannot be generalized to mothers attending private hospitals or clinics, mothers delivering baby at home, and mothers living in areas other than the study area. Second, being a case control study, the study participants may have been subjected to recall bias. The third limitation of this study was we could not assess the relationship between weight gain during pregnancy and risk of LBW as data related to maternal weight during regular intervals of pregnancy was not available in our study. However, we took maternal weight during first trimester as a proxy for prepregnancy maternal weight which could have been a better measure for assessing LBW [41].

## 5. Conclusion

Educational and occupational status, mode of delivery, gestational age, maternal weight, and history of LBW were found to be independent predictors of LBW. There is need of developing coordination with education sector for increasing educational status of mothers and adolescent girls. Social determinants of health need to be considered while developing interventional programs. Similarly, interventional programs need to be developed considering identified predictors of low birth weight.

## Figures and Tables

**Table 1:** Sociodemographic characteristics of study participants, Lumbini Provincial Hospital, 2019.

Variables	Category	Frequency	Percentage
Age group of respondent	<20	18	5.7
	20–30	227	72.1
	>30	70	22.2

Ethnicity of respondent	Dalit	36	11.4
	Janjati	79	25.1
	Religious minorities	31	9.8
	Upper caste	169	53.7

Educational status of respondent	Literate	281	89.2
	Illiterate	34	10.8

Occupation of respondent	Labor/wage worker	17	5.4
	Unemployed/wousewife	250	79.4
	Employed	48	15.2

Family type of respondent	Nuclear	188	59.7
	Joint	127	40.3

**Table 2:** Maternal and obstetric characteristics of study participants, Lumbini Provincial Hospital, 2019.

Variables	Category	Frequency	Percentage
Mode of delivery	Cesarean section	198	62.9
	Vaginal	117	37.1

Gestational age	<37 weeks	48	15.2
	≥37 weeks	267	84.8

Parity	Primiparity	161	51.1
	Multiparity	154	48.9

History of abortion	Yes	28	8.9
	No	287	91.1

History of low birth weight	Yes	24	7.6
	No	291	92.4

Maternal weight	<50 kilograms	75	23.8
	≥50 kilograms	240	76.2

ANC visit	≥4 visit	251	79.7
	<4 visit	64	20.3

Hb level	<11 g/dl	243	77.1
	≥11 g/dl	72	22.9

Rest during day time	≤2 hours	276	87.6
	>2 hours	39	12.4

Rest during night time	<8 hours	253	80.3
	≥8 hours	62	19.7

**Table 3:** Sociodemographic predictors of LBW.

Variables	Cases (%) (*n* = 105)	Control (*n* = 210)	COR	95% CI	AOR	95% CI
Mothers age group						
<20	11 (10.5)	7 (3.3)	3.45	1.28–9.27^*∗*^	2.71	0.93–7.94
20–30	71 (67.6)	156 (74.3)	1		1	
>30	23 (21.9)	47 (22.4)	1.07	0.60–1.90	1.18	0.63–2.21

Respondent ethnicity						
Dalit	19 (18.1)	17 (8.1)	2.51	1.21–5.22^*∗*^	1.77	0.79–3.92
Janjati	21 (20.0)	58 (27.6)	0.81	0.44–1.47	0.62	0.32–1.19
Religious minorities	13 (12.4)	18 (8.6)	1.62	0.74–3.56	0.44	0.15–1.30
Upper caste	52 (49.5)	117 (55.7)	1		1	

Respondent educational status						
Literate	83 (79)	198 (94.3)	0.22	0.10–0.48^*∗*^	0.32	0.13–0.81^*∗∗*^
Illiterate	22 (21)	12 (5.7)	1		1	

Occupation of respondent						
Labor/wage worker	11 (10.5)	6 (2.9)	9.16	2.62–32.03^*∗*^	5.21	1.13–23.88^*∗∗*^
Unemployed/housewife	86 (81.9)	164 (78.1)	2.62	1.17–5.85	2.63	1.11–6.20
Employed	8 (7.6)	40 (19)	1		1	

Family type of respondent						
Nuclear	70 (66.7)	118 (56.2)	1.55	0.95–2.54		
Joint	35 (33.3)	92 (43.8)	1			

Wealth index						
Poorest	32 (30.5)	31 (14.8)	2.58	1.23–5.39^*∗*^	1.71	0.73–4.03
Poorer	18 (17.1)	44 (21.0)	1.02	0.47–2.21	0.97	0.42–2.22
Middle	21 (20.0)	43 (20.5)	1.22	0.57–2.59	1.19	0.54–2.62
Richer	16 (15.2)	47 (22.4)	0.851	0.38–1.87	0.91	0.40–2.07
Richest	18 (17.1)	45 (21.4)	1			

^∗^Variables entered to multivariate regression model, ^∗∗^Statistically significant at p-value <0.05, adjusted model.

**Table 4:** Maternal and obstetric predictors of LBW.

Variables	Cases (%) (*n* = 105)	Control (%) (*n* = 210)	COR	95% CI	AOR	95% CI
Mode of delivery						
Cesarean section	58 (55.2)	59 (28.1)	3.15	1.93–5.14^*∗*^	2.19	1.21–3.97^*∗∗*^
Vaginal	47 (44.8)	151 (71.9)	1		1	

Gestational age						
<37 weeks	31 (29.5)	17 (8.1%)	4.75	2.48–9.10^*∗*^	2.51	1.15–5.48^*∗∗*^
≥37 weeks	74 (70.5)	193 (91.9%)	1			

Parity						
Primi	49 (46.7)	112 (53.3)	0.76	0.47–1.22		
Multi	56 (53.3)	98 (46.7)	1			

History of abortion						
Yes	10 (9.5)	18 (8.6)	1.12	0.49–2.52		
No	95 (90.5)	192 (91.4)	1			

History of low birth weight						
Yes	17 (16.2)	7 (3.3)	5.60	2.24–13.98^*∗*^	5.12	1.93–13.60^*∗∗*^
No	88 (83.8)	203 (96.7)	1			

Maternal weight						
<50 kilograms	34 (32.4)	41 (19.5)	1.97	1.15–3.36^*∗*^	2.23	1.23–4.02^*∗∗*^
≥50 kilograms	71 (67.6)	169 (80.5)	1			

ANC visit						
≥4 visits	74 (70.5)	177 (84.3)	0.44	0.25–0.77^*∗*^	0.61	0.32–1.16
<4 visits	31 (29.5)	33 (15.7)	1			

Hb level						
<11 g/dl	91 (86.7)	152 (72.4)	2.48	1.30–4.69^*∗*^	1.79	0.89–3.57
≥11 g/dl	14 (13.3)	58 (27.6)	1			

Rest during day time						
≤2 hours	97 (92.4)	179 (85.2)	2.10	0.92–4.74		
>2 hours	8 (7.6)	31 (14.8)	1			

Rest during night time						
<8 hours	92 (87.6)	161 (76.7)	2.15	1.11–4.18^*∗*^	1.74	0.83–3.63
≥8 hours	13 (12.4)	49 (23.3)	1			

^∗^Variables entered to multivariate regression model, ^∗∗^Statistically significant at p-value <0.05, adjusted model.

## Data Availability

The data used to support the findings of this study are available from the corresponding author upon request.

## Abbreviations

ANC: Antenatal care
AOR: Adjusted odds ratio
CI: Confidence interval
COR: Crude odds ratio
LBW: Low birth weight
WHO: World Health Organization.
